# Mothers’ Nutrition Knowledge Is Unlikely to Be Related to Adolescents’ Habitual Nutrient Intake Inadequacy in Japan: A Cross-Sectional Study of Japanese Junior High School Students

**DOI:** 10.3390/nu12092801

**Published:** 2020-09-13

**Authors:** Mai Matsumoto, Yoichi Hatamoto, Ayumi Masumoto, Azusa Sakamoto, Shinji Ikemoto

**Affiliations:** 1 Department of Nutritional Epidemiology and Shokuiku, National Institutes of Biomedical Innovation, Health, and Nutrition, 1-23-1 Toyama, Shinjuku-ku, Tokyo 162-8636, Japan; m-matsumoto@nibiohn.go.jp; 2 Department of Nutrition and Metabolism, National Institutes of Biomedical Innovation, Health, and Nutrition, 1-23-1 Toyama, Shinjuku-ku, Tokyo 162-8636, Japan; yhatamoto@nibiohn.go.jp; 3 Saitama City, 6-4-4 Tokiwa, Urawa-ku, Saitama-shi, Saitama 330-9588, Japan; ayumimasumoto311@hotmail.com; 4 Department of Registered Dietitian, HANA College of Nutrition, 1-1-12 Negishi, Taitou-ku, Tokyo 110-8662, Japan; azusa_sakamoto_j@yahoo.co.jp; 5 Department of Human Nutrition, Seitoku University, 550 Iwase, Matsuo-shi, Chiba 271-8555, Japan

**Keywords:** nutrition knowledge, mother, adolescence, nutrition adequacy

## Abstract

Dietary habits in adolescence persist into adulthood; thus, it is important to identify the factors that influence adolescent diet and establish a healthy diet. This study aimed to examine the association between mothers’ nutrition knowledge and their children’s nutrient intake inadequacy among Japanese junior high school student–mother dyads. The participants were 288 students and their mothers. Data regarding mothers’ nutrition knowledge were obtained using a validated, self-administered general nutrition knowledge questionnaire for Japanese adults (JGNKQ). Participants were categorised into two groups according to the mothers’ total JGNKQ scores. Adolescents’ dietary habits during the preceding month were assessed using a brief self-administered diet history questionnaire. Inadequacy of each nutrient intake was assessed using the cut-point method, which showed that 14 nutrients were below “estimated average requirement (EAR)” and five nutrients were outside the range of “tentative dietary goal to prevent lifestyle-related diseases (DG)”. In the habitual daily nutrient intakes and the proportion of nutrient intake inadequacy of the students, no differences were observed according to mother’s nutritional knowledge level. Our findings suggest that mothers’ nutrition knowledge may not be directly associated with adolescents’ nutrient intake among Japanese junior high school student–mother dyads.

## 1. Introduction

Obesity is a serious clinical and public health problems worldwide [1]. The proportion of obesity has been reported to increase during adolescence for young adults in developed countries, including Japan [2,3,4]. Dietary habits have been reported to have a great influence on the development of lifestyle-related diseases, such as obesity and diabetes [5]. Diet and dietary behaviours which develop during adolescence tend to persist throughout life [6,7,8], and these dietary habits may be difficult to change later in life [9]. Therefore, it is important to identify the factors that influence adolescent diet to establish the healthy eating patterns which may lead to prevention of chronic diseases including obesity [10].

The family environment, such as parent role and attitudes and food availability, social environment, including peers and their lifestyle, are associated with the dietary behaviour of adolescents, unlike those of early childhood and school-age [11,12]. Especially, junior high school students in early adolescence are at the beginning of a transitional period from child to adult and various factors, including not only themselves but also their parents and society, may affect their diet [13]. Nutrition knowledge is one of the factors that influence diet and predict change in dietary intake [14]. For example, parents’ nutrition knowledge are reportedly related to their preschool or elementary school children’s healthy diet, including vegetable and fruits intakes [15,16,17,18]. However, no studies about the relation between parents’ nutrition knowledge and the adolescent’s diet have reported. Nutrition education to improve diet should ideally be implemented after appraising the level of nutrition knowledge of those targeted [19]. Therefore, the revelation of the association between parents’ nutrition knowledge and the adolescent’s diet is important from the viewpoint of whether the education should be targeted to the adolescent or the adolescent plus their parents. Several studies reported that mothers have a greater influence on children’s diet than fathers [20,21]. Therefore, we examined the association between mothers’ general nutrition knowledge and inadequacy of their children’s nutrient intake among Japanese junior high school student–mother dyads.

## 2. Methods

### 2.1. Study Design and Participants

A self-administered paper anonymous diet history questionnaire [22] was distributed by teachers to 851 junior high school students aged 12 to 15 years in all classes (boys; *n* = 402, girls; *n* = 449) and a set of two self-administered paper questionnaires (Japanese general nutrition knowledge questionnaire (JGNKQ) [23] and lifestyle questionnaire) was distributed by those students to their mothers in Saitama city, Kanto region, Japan in June 2017. The school was selected by a staff member of Saitama city and provided school lunches to all participants. Additionally, no mother had eligible brothers and sisters among participants. The questionnaires were sealed in an envelope and collected in a designated collection box installed in the school. The completed questionnaires were examined by research staff, and those with missing information were returned to the students or mothers by mail for completion. At the research office, the postal staff members who did not check the questionnaires and data received the envelopes in a sealed state and mailed it using the connectable correspondence table (ID and information such as name and address). We asked participants to send the completed questionnaire back to the research office by mail. Overall, 427 student–mother dyads completed the questionnaires. We excluded students with missing information on the variables used (*n* = 70), and students (*n* = 23) with a reported energy intake less than half the energy requirement for the lowest physical activity category according to the Japanese Dietary Reference Intake (DRIs) (boys: <1150 kcal/day and girls: <1075 kcal/day) or ≥1.5 times the energy requirement for the highest physical activity category (boys: ≥4350 kcal/day and girls: ≥4050 kcal/day), which were judged as miss-reporters [24,25]. Additionally, we excluded mothers with missing information on at least one of the variables used in the present analysis (*n* = 47). Thus, the final participant numbers were 287 student–mother dyads.

Written informed consent was obtained from all students and mothers. This study was conducted according to the guidelines laid down in the Declaration of Helsinki, and all procedures involving human subjects were approved by the Ethics committee of Seitoku University (approval number H28U045).

### 2.2. Assessment of Mothers’ Nutrition Knowledge Level

Nutrition knowledge level was assessed using a previously validated Japanese general nutrition knowledge questionnaire for adults aged 18–64 years (JGNKQ) [23]. Details of the JGNKQ’s structure, validity, and reliability have been published elsewhere [23]. Briefly, the JGNKQ is a self-administered 10-page questionnaire including five sections, i.e., “Dietary recommendations” (Section 1; 9 items); “Sources of nutrients” (Section 2; 96 items); “Choosing everyday foods” (Section 3; 5 items); “Diet-disease relationships” (Section 4; 20 items); and “Reading a food label” (Section 5; 17 items); with a total of 147 items. In a study of 1182 Japanese adults aged 18–64 years, the Cronbach’s α value of internal reliability of each section ranged between 0.31 and 0.94, and that of the overall questionnaire was 0.95. Additionally, a study that assessed construct validity among 96 female undergraduate Japanese students in a nutrition major and 44 female undergraduate Japanese students in other majors by self-administered questionnaire showed that students in a nutrition major consistently scored higher than students in other majors on all five sections of the questionnaire (*p* = 0.000) [23]. Moreover, the test–retest (during 2 weeks) reliability correlation coefficients for each section ranged from 0.44 to 0.68 and the overall correlation coefficient was 0.75 in a study of 75 female undergraduate Japanese students. Among the 75 female university students, there were no significant differences between the first and second test scores, thus, systematic differences were not observed [23].

JGNKQ is a multiple choices questionnaire (three to five items, and choices including “I don’t know), and the responses were converted to binary numbers, with 1 and 0 representing correct and incorrect answers, respectively. Therefore, the maximum score on the 147-item questionnaire was 147 points. A higher nutrition knowledge score reflects a higher nutrition knowledge level. In the current study, we classified participants into two nutrition knowledge level groups by median split the total score of JGNKQ: low (<80 points) and high nutrition knowledge groups.

### 2.3. Dietary Assessment

Habitual dietary intake during the preceding month was assessed using a brief self-administered diet history questionnaire for Japanese children and adolescents (BDHQ15y) [22]. BDHQ15y was developed based on the adult version of the validated brief self-administered diet history questionnaire for Japanese adults (BDHQ) [26,27]. BDHQ15y is a four-page structured questionnaire comprising 67 questions on the frequency of intake of food items commonly cooked and consumed in Japan, except supplements. Daily food, energy and selected nutrient intakes were calculated using an ad-hoc computer algorithm for BDHQ15y [22], based on the Standard Tables of Food Composition in Japan [28]. We could not include intake from dietary supplements in the analysis because reliable composition tables of dietary supplements were lacking in Japan. The validity of BDHQ15y was verified by a study on the relationship between selected food intake and blood biomarker levels [22]. Any self-estimated dietary assessments cannot avoid under- or over-reporting of dietary intake [24,25]. Based on a previous study, physical activity level was defined to level II in all students, due to absence of quantitative information about physical activity [29,30,31]. Thus, the following calculation/equation was applied to adjust reported nutrient intakes in order to compare reported nutrient intakes with Japanese DRI values, except fat and carbohydrate: adjusted nutrient intake = reported intake/reported energy intake x EER (estimated energy requirement). The percentage of daily energy intake was calculated using the crude value for total fat and carbohydrate intake. Additionally, food intake values were energy adjusted using the density method (i.e., the percentage of energy for energy-providing nutrients and their amounts per 1000 kcal for food groups) to minimise the influence of dietary misreporting.

### 2.4. Determination of Habitual Nutrient Intake Inadequacy

Inadequate intake of each nutrient was determined by comparing nutrient levels with each dietary reference value according to the Japanese DRIs using a previously reported method [29,30,31]. “Estimated average requirement (EAR)” is set to avoid insufficient intake of nutrients, whereas “tentative dietary goal to prevent lifestyle-related diseases (DG)” is set to prevent non-communicable diseases. The intake level below EAR was considered as inadequate using the cut-point method for the following 14 nutrients with known EARs: protein, vitamin A expressed as retinol activity equivalents, vitamin B_1_, vitamin B_2_, niacin expressed as niacin equivalent, vitamin B_6_, vitamin B_12_, folate, vitamin C, calcium, magnesium, iron, zinc, and copper [32]. For the following five nutrients, the intake level outside the range of DG values was considered as not meeting the standard DG: total fat, carbohydrate, total dietary fibre, sodium expressed as salt-equivalent, and potassium [32].

### 2.5. Other Variables

Using the BDHQ15y, children were asked about their age, body weight and height, and use of dietary supplements. The body mass index (BMI) was calculated as weight (kilograms) divided by the square of height (meters) because self-reported height and weight in adolescence have been reported to be able to assess at the population level in epidemiological study [29,30,31]. Obesity and thinness were determined using the definition of the reports by Cole et al. [33,34]. In the BDHQ15y, children also reported the frequency of exercise, such as sports club activities per week for the past month (everyday, 4–6 days/week, 2–3 days/week, 1 day/week, or never) as habitual exercise.

Mothers reported their age, educational background (junior high school or high school, junior college or vocational technical school, or university or higher), household income (less than 2 million yen/year, 2 million to 6 million yen/year, 6 million to 10 million yen/year, or more than 10 million yen/year), working status (full-time, part-time, housewife, or others), marital status (yes or no), number of children (one, two, or more than three), and frequency of dinner cooking per week in the lifestyle questionnaire.

### 2.6. Statistical Analysis

All statistical analyses excluding sample size calculation were performed using the IBM SPSS statistics software package (version 22.0, SPSS Inc., Chicago, IL, USA). All reported *p*-values were two-tailed, with a *p* value of 0.05 considered statistically significant. The differences in characteristics among the low and high nutrition knowledge groups were compared using the chi-square test for categorical variables and an independent t-test for continuous variables. Gender and mothers’ educational level which were found to be significantly different (*p* < 0.05) between groups categorized by nutrition knowledge level were considered as potential confounding factors in the first model. Additionally, mother’s age, household income, mother’s working status, frequency of mother’s dinner cooking, and the number of children in home, which were reported to be related mothers’ nutrition knowledge or adolescents’ dietary intake, were added into confounding variables in the second model [19,24,35,36,37]. The mean intake of nutrients and food groups among junior high school students as dependent variables (continuous variables) was compared using a covariate analysis (ANCOVA) with mother’s nutrition knowledge level (high or low), gender and mothers’ educational level in the first model and further added mother’s age, household income, mother’s working status, frequency of mother’s dinner cooking and the number of children in the second model as an independent variable. The logistic regression analysis was used to examine adjusted odds ratio and their 95% confidence interval for nutrition inadequacy as the dependent variable (categorical variable), with mother’s nutrition knowledge level (high or low), gender and mothers’ educational level in the first model and further added mother’s age, household income, mother’s working status, frequency of mother’s dinner cooking, and the number of children in the second model as independent variables. The number of participants required to detect the middle effect size (f = 0.25), which is the default value when you do not know the effect size, with a significance level of 0.05 and statistical power of 0.8 in ANCOVA was estimated to be 128 in total, according to the power analysis using G*Power 3 [38]. Additionally, the number of participants in the logistic regression analysis with the incidence of nutrition inadequacy (0.15), a significance level of 0.05 and statistical power of 0.8, was estimated to be 199 in total, according to the power analysis using G*Power 3 [38]; thus, the number of participants in the present study was enough to evaluate the statistically significant difference.

## 3. Results

Basic characteristics of participants are shown in Table 1. Total nutrition knowledge score (mean ± standard deviation (SD)) of the high and low nutrition knowledge groups were 94.4 ± 10.6 and 59.4 ± 17.3, respectively, and significant difference was observed between two groups (*p* < 0.001). The proportion of girls in high group was higher than those of low group (*p* = 0.041). No significant difference was observed in BMI (mean ± SD) among children between two groups (High: 18.4 ± 2.3 kg/m^2^, Low: 18.5 ± 2.4 kg/m^2^). In addition, the energy intake (mean ± SD) for children was not different between high and low groups (High: 2377 ± 685 kcal/day, Low: 2432 ±704 kcal/day). Moreover, mothers in the Low group had a higher proportion of high school or junior high school (high: 7.5%, low: 18.4%) and a lower proportion of university or higher educational level (high: 40.4%, low: 36.9%) compared to the high group. The age of both children and mothers, number of days exercising for children, household income, working status, marital status, and number of children for mothers and frequency of mothers’ dinner cooking also observed no differences between the high and low nutrition knowledge groups. In addition, the percentage of students that used supplements, such as calcium, iron, and vitamins, was approximately 10% in this study (data not shown), and there is no difference between two groups.

The habitual daily nutrient intake and inadequate intake of each nutrient among 287 junior high school students are shown in Table 2. The daily intakes of most nutrients tended to be higher in the high nutrition knowledge group than the low nutrition knowledge group, although the differences were not significant. For nutrient intake inadequacy, no significant difference was also observed according to mothers’ nutrition knowledge level.

Table 3 shows the habitual daily food groups intake among students according to mothers’ nutrition knowledge level. The only bread intake in high group (17.3 ± 12.1 g/1000 kcal) was lower than those of low group (20.7 ± 14.6 g/1000 kcal) after adjusted for confounding factors (*p* = 0.009).

## 4. Discussion

The present study evaluated the association between junior high school students’ nutrient inadequacy with their mothers’ nutrition knowledge level. We found that the nutrients intake and inadequacy were not significantly different among junior high school students categorised according to their mothers’ nutrition knowledge level. To the best of our knowledge, there were few studies about the relationship between mothers’ nutrition knowledge and nutrient intake inadequacy among junior high school students, especially using a validated nutrition knowledge questionnaire.

In the current study, the proportion of girls in high groups was higher compared to low groups. The response rate of boy–mother dyads and girl–mother dyad was 32.1% and 35.2%, respectively. The nutrition knowledge questionnaire used in the present study consists of 10 pages, and it takes 20 to 30 min to answer. If the participants were not interested in health or nutrition, they may refuse to answer. Thus, the mothers of girls might have been highly health conscious and have higher nutrition knowledge level than those of boys. However, the results of analysis of nutrients and food groups intakes by gender were similar to those of all participants (data not shown). The only educational level of mothers differed according to nutrition knowledge level between the high and low nutrition knowledge groups. A study on nutrition knowledge among middle-aged Belgian women reported that age, educational level, and working status affect nutrition knowledge [35]. Our result was inconsistent with this previous study with respect to age and working status. The participants in this previous study were 18–39 years old and more than half of these had no children, while the participants in the present study were limited to mothers of junior high school students, and the age range was narrow (39–50 years). Thus, these background differences might have influenced the differences in results between the previous study and the present study.

Regarding daily nutrient intake, the habitual intake of all nutrients by students was not different after adjustment for confounding factors. In the Japanese study, it was reported that maternal nutrition knowledge level is related to the intake of healthy food, such as vegetables, for early elementary school children, but the tendency has been to weaken in the upper grades of elementary school [18]. Thus, mother’s nutrition knowledge may be less affected as the child ages. In fact, the only bread intake was observed in the difference according to mother’s nutrition knowledge level, and no difference was found in other food groups intakes, including vegetable and fruits. All cereals intake was also not different between two groups in the present study. The selection of staple food may be different according to mother’s nutrition knowledge. However, it may be less likely to relate to cereals and nutrients intakes, because rice is the main food as a staple food among Japanese [39].

The nutrients intake inadequacy of students was not also different according to mothers’ nutrition knowledge level in the present study. Among participants in this study, the proportion of adolescents with inadequacy of vitamin B_1_, iron, total dietary fibre and sodium was high at approximately 80%, 65%, 85%, and 95%, respectively. These results were consistent with the results of the previous study for Japanese junior high school students [40,41]. In other words, the results of present study were not specific results, and it may be the data that show the tendency of Japanese junior high school students. It has been reported that television, newspapers, and the internet are the main sources of food information among adolescents, whereas parents are the main source of food information for children aged 9 to 11 years [42,43]. Additionally, in a US study, it was reported that elementary school students’ nutrition knowledge did not affect their diet; however, it was related to their own nutrition knowledge and food selection in junior high school students [44]. Concerning the differences between children and adolescents, it has been reported that adolescents are affected by society, while elementary school children are more affected by their family [45]. Factors determining the eating behaviour of adolescents also included money, convenience, gender, friends, and the environment [46,47]. Furthermore, it has been reported that adolescence is a period of increased responsibility and autonomy [46,47]. In addition, the preschool and elementary children had the highest overall diet quality than adolescents [48] Given these facts, there is the possibility that this period, among junior high school students, might be the beginning of the stage when adolescents start to become unaffected by parental nutrition knowledge.

Several limitations of this study need to be mentioned. First, the participants’ selection from a school in Kanto urban region in Japan was not a random sample selection from the general population. The participants are therefore not likely to be representative of all Japanese junior high school students and their mothers. However, the height and weight of the participants in the present study were similar to the representative values in Japan [49]. Additionally, the household income of the participants in the current study was similar to those families with more than one child in Japan, and the working status of the mothers was similar to the representative value of Japan [50]. Second, the diet of adolescents may be influenced by their own nutrition knowledge. Indeed, parents’ nutrition knowledge was reported to affect children’s diet until elementary school age [18]; it has also been reported that Iranian female high school students with high nutrition knowledge have a healthy diet [51]. Unfortunately, this research did not evaluate nutrition knowledge of junior high school students. Therefore, it cannot be denied that this may have influenced the results of the current study. However, there is no validated questionnaire that can evaluate the nutrition knowledge of junior high school students in Japan. Additionally, we did not assess the gender of children other than the participants in their home. Finally, we used BDHQ15y to assess the dietary intake; however, its ability to estimate dietary intakes was validated on limited foods and nutrients.

## 5. Conclusions

The results of this study showed that habitual nutrient intake adequacy of Japanese junior high school students was not related to their mothers’ nutrition knowledge level. Our findings suggest that mother’s nutrition knowledge should not directly be targeted to improve adolescents’ diet and nutrition status. Therefore, further studies are needed to determine alternative factors that could possibly influence dietary intake among Japanese adolescents.

## Figures and Tables

**Table 1:** Characteristics of study participants categorized into high and low groups by mother’s nutrition knowledge level.

	High (*n* = 146)		Low (*n* = 141)		*p*
Mother’s nutrition knowledge score, Mean SD	94.4	10.6	59.4	17.3	<0.001
Children					
Gender, *n*, %					0.041
Boys	57	39.0	72	51.1	
Girls	89	61.0	69	48.9	
Age (years), Mean, SD	13.2	1.0	13.0	1.0	0.159
Body height (cm), Mean, SD	156.1	8.3	156.6	8.7	0.661
Body weight (kg), Mean, SD	45.1	7.8	45.7	8.8	0.508
Body mass index (kg/m^2^), Mean, SD	18.4	2.3	18.5	2.4	0.669
Body mass index, *n*, %					0.108
Thinness	24	16.4	19	13.5	
Normal	119	81.5	112	79.4	
Obesity	3	2.1	10	7.1	
Energy intake (kcal/day), Mean, SD	2377	685	2432	704	0.499
Number of days exercising, *n*, %					0.880
Everyday	84	57.5	77	54.6	
4–6 days/week	30	20.5	33	23.4	
2–3 days/week	9	6.2	12	8.5	
1 day/week	8	5.5	7	5.0	
Never	15	10.3	12	8.5	
Mother					
Age (years), *n*, %					0.434
Less than 40 years	7	4.8	12	8.5	
40–49 years	118	80.8	108	76.6	
50 years or over	21	14.4	21	14.9	
Education level, *n*, %					0.022
University or higher	59	40.4	52	36.9	
Junior college or vocational technical school	76	52.1	63	44.7	
High school or junior high school	11	7.5	26	18.4	
Annual household income, *n*, %					0.222
Less than 2,000,000 yen	8	5.5	6	4.3	
2,000,000 yen to 6,000,000 yen	16	11.0	22	15.6	
6,000,000 yen to 10,000,000 yen	61	41.8	69	48.9	
10,000,000 yen or more	61	41.8	44	31.2	
Working status, *n*, %					0.936
Full-time	19	13.0	21	14.9	
Part-time	74	50.7	73	51.8	
Housewife	48	32.9	42	29.8	
Others	5	3.4	5	3.5	
Marriage status, *n*, %					0.955
Yes	141	96.6	136	96.5	
No	5	3.4	5	3.5	
Number of children, *n*, %					0.756
1	29	19.9	33	23.4	
2	91	62.3	83	58.9	
3 or more	26	17.8	25	17.7	
Frequency of dinner cooking per week, Mean, SD	6.7	0.7	6.6	0.9	0.088

SD, standard deviation; The *p* values are shown for chi-square test for categorical variables and for independent t test for continuous variables between high and low groups among boys or girls or their mothers.

**Table 2:** Habitual daily nutrient intakes and prevalence of not meeting estimated average requirement (EAR) or preventing lifestyle-related disease(DG) of Dietary Reference Intakes(DRIs) among 287 junior high school students categorized into high and low groups by mothers’ nutrition knowledge level ^†^.

	High (*n* = 146)		Inadequacy ^‡^ (%)	Low (*n* = 141)		Inadequacy ^‡^ (%)	*p* ^¶^	*p* ^††^	OR (95%CI) ^‡‡^			OR (95%CI) ^§§^		
	Mean	SD		Mean	SD									
Nutrient with EAR														
Protein (g)	83	15	0	82	14	0	0.563	0.695						
Vitamin A (µgRAE) ^§^	815	395	17.8	768	279	20.6	0.363	0.418	0.888	(0.483)	(1.632)	0.970	(0.504)	1.868
Vitamin B_1_ (mg)	1.0	0.17	80.1	1.0	0.16	86.5	0.443	0.648	0.646	(0.338)	(1.235)	0.712	(0.358)	1.418
Vitamin B_2_ (mg)	1.8	0.5	8.9	1.8	0.4	7.1	0.900	0.775	1.389	(0.570)	(3.388)	1.443	(0.547)	3.803
Niacin (mgNE) ^ǁ^	17.3	4.3	8.9	16.7	4.0	9.2	0.248	0.295	1.046	(0.454)	(2.410)	0.950	(0.397)	2.273
Vitamin B_6_ (mg)	1.4	0.31	18.4	1.4	0.28	25.5	0.249	0.385	0.676	(0.376)	(1.215)	0.686	(0.369)	1.275
Vitamin B_12_ (mg)	8.8	4.3	0	8.2	3.8	1.4	0.306	0.327		-				
Folate (µg)	391	128	4.3	368	105	1.4	0.137	0.226	3.577	(0.667)	(19.176)	3.571	(0.592)	21.54
Vitamin C (mg)	131	53	13.7	125	47	18.4	0.374	0.586	0.760	(0.396)	(1.460)	0.856	(0.431)	1.700
Calcium (mg)	905	312	34.9	924	301	34.0	0.654	0.569	1.106	(0.664)	(1.841)	1.192	(0.695)	2.047
Magnesium (mg)	302	60	15.8	295	51	16.3	0.154	0.246	0.911	(0.477)	(1.738)	0.882	(0.441)	1.762
Iron (mg)	8.9	2.0	63.0	8.4	1.8	67.4	0.057	0.105	0.675	(0.398)	(1.147)	0.697	(0.398)	1.220
Zinc (mg)	10.4	1.5	0.7	10.3	1.5	2.8	0.273	0.351	0.276	(0.029)	(2.591)	0.292	(0.023)	3..670
Copper (mg)	1,31	0.20	0	1.28	0.21	0	0.045	0.086		-				
Nutrient with DG														
Fat (%energy)	30.0	8.6	50.7	30.6	9.6	51.8	0.480	0.564	0.928	(0.572)	(1.506)	0.970	(0.588)	1.602
Carbohydrate (%energy)	57.6	18.8	30.1	58.7	19.9	30.5	0.927	0.750	1.004	(0.598)	(1.683)	1.084	(0.632)	1.860
Total dietary fiber (g)	13.2	3.3	86.3	12.8	3.4	86.5	0.262	0.448	1.007	(0.504)	(2.012)	1.205	(0.576)	2.521
Sodium (salt-equivalent) (g)	11.9	2.6	97.9	11.7	2.5	94.3	0.303	0.347	2.470	(0.628)	(9.712)	2.057	(0.443)	9.555
Potassium (mg)	2877	716	29.5	2801	593	29.1	0.329	0.521	1.066	(0.631)	(1.800)	1.182	(0.671)	2.082

DG, tentative dietary goal for preventing lifestyle-related disease; DRIs, Dietary Reference Intakes; EAR, estimated average requirement; OR, odds ratio; SD, standard deviation; 95%CI, 95% Confidence Interval; ^†^ Adjustment of reporting error was performed according to the following: Nutrient intake = reported nutrient intake / reported energy intake × estimated energy requirement. The estimated energy requirement of physical activity level Ⅱ for 12- to 14-year old Japanese boys and girls are 2600 kcal/day and 2400 kcal/day, respectively; ^‡^ Percentage of participants whose nutrient intake did not meet DG or EAR of DRIs. Each nutrient intake was compared with each DRI value, using the cut-point methods; ^§^ Sum of retinol, β-carotene/12, α-carotene/24, and cryptoxanthin/24; ^ǁ^ Sum of niacin and protein/6000; ^¶^ The *p* values are shown for covariate analysis to analyze difference of nutrient intakes between high and low groups adjusted for confounding variables of gender and mother’s education level (university or high, Junior college or vocational technical school and High school or, junior high school); ^††^ The *p* values are shown for covariate analysis to analyze difference of nutrient intakes between high and low groups adjusted for confounding variables of gender, mother’s education level (university or high, Junior college or vocational technical school and High school, or junior high school), mother’s age (less than 40 years, 40–49 years, or 50 years or over), household income (less than 2,000,000 yen, 2,000,000–6,000,000 yen, 6,000,000–10,000,000 yen, or 10,000,000 yen or more), mother’s working status (full-time, part-time, housewife, or others), frequency of mother’s dinner cooking and the number of children in home (one, two, or three or more); ^‡‡^ Multivariate adjusted ORs about nutrient intake inadequacy between high and low groups were calculated by adjusting for gender and mother’s education level (university or high, Junior college or vocational technical school and High school, or junior high school); ^§§^ Multivariate adjusted ORs about nutrient intake inadequacy between high and low groups were calculated by adjusting for gender, mother’s education level (university or high, Junior college or vocational technical school and High school, or junior high school), mother’s age (less than 40 years, 40–49 years, or 50 years or over), household income (less than 2,000,000 yen, 2,000,000–6,000,000 yen, 6,000,000–10,000,000 yen, or 10,000,000 yen or more), mother’s working status (full-time, part-time, housewife, or others), frequency of mother’s dinner cooking and the number of children in home (one, two, or three or more).

**Table 3:** Habitual daily food group intakes among 287 junior high school students categorized into high and low groups by mother’s nutrition knowledge level (g/1000 kcal).

	High (*n* = 146)		Low (*n* = 141)		*p* ^‡^	*p* ^§^
	Mean	SD	Mean	SD		
Cereals	222.4	67.8	220.2	70.2	0.316	0.237
Rice	180.3	72.2	173.3	73.5	0.119	0.077
Bread	17.3	12.1	20.7	14.6	0.017	0.009
Noodles	24.8	14.9	26.2	16.6	0.489	0.513
Pulses	27.9	19.5	26.7	17.1	0.727	0.896
Potatoes	14.9	10.5	13.8	9.4	0.510	0.666
Sugar	2.3	2.0	2.3	2.4	0.701	0.626
Confectioneries	43.6	25.1	40.9	25.3	0.525	0.573
Fat and oil	7.3	3.5	7.0	3.0	0.671	0.666
Fat	0.6	0.8	0.6	0.8	0.747	0.851
Oil	6.7	3.3	6.4	2.8	0.718	0.684
Fruits	31.1	27.6	34.3	32.6	0.275	0.166
Total vegetables	113.4	60.5	101.4	55.3	0.184	0.275
Green and yellow vegetables	43.1	27.4	39.2	24.2	0.406	0.542
Other vegetables	56.4	33.2	50.0	29.0	0.198	0.301
Pickled vegetables	4.7	6.9	4.2	5.3	0.239	0.255
Mushrooms	4.0	3.8	3.5	3.3	0.371	0.368
Seaweeds	5.2	5.1	4.4	4.1	0.284	0.315
Beverages	294.1	169.6	287.8	169.3	0.999	0.965
Fruit and vegetable juice	30.3	46.8	27.2	47.3	0.539	0.595
Green tea	185.0	133.8	167.8	124.4	0.488	0.511
Black tea	31.7	66.8	26.6	60.6	0.726	0.647
Soft drinks	47.2	66.6	66.2	81.0	0.058	0.075
Fish and shellfish	29.8	17.5	26.9	16.3	0.257	0.262
Meat	37.4	18.7	36.2	16.8	0.798	0.823
Eggs	16.1	10.0	15.5	10.6	0.960	0.940
Dairy products	142.7	97.0	155.8	104.4	0.268	0.275

SD, standard deviation; Adjustment of reporting error was performed according to the following: Food group intake = reported food group intake/reported energy intake × 1000(kcal); ^‡^ The *p* values are shown for covariate analysis to analyze difference of nutrient intakes between high and low groups adjusted for confounding variables of gender and mother’s education level (university or high, Junior college or vocational technical school and High school, or junior high school); ^§^ The *p* values are shown for covariate analysis to analyze difference of nutrient intakes between high and low groups adjusted for confounding variables of gender, mother’s education level (university or high, Junior college or vocational technical school and High school, or junior high school), mother’s age (less than 40 years, 40–49 years, or 50 years or over), household income (less than 2,000,000 yen, 2,000,000–6,000,000 yen, 6,000,000–10,000,000 yen, or 10,000,000 yen or more), mother’s working status (full-time, part-time, housewife, or others), frequency of mother’s dinner cooking and the number of children in home (one, two, or three or more).
